# The effectiveness of portable ultrasound-guided resuscitative endovascular balloon occlusion of the aorta for stopping iliac artery hemorrhage during first aid pre-hospital: a randomized control animal trial

**DOI:** 10.1007/s00068-022-01895-1

**Published:** 2022-04-12

**Authors:** Yuqing Huang, Haiyan Kou, Yuhao Kong, Xuexia Shan, Shengzheng Wu, Xianghui Chen, Xingxi Lin, Liye Zhang, Faqin Lv, Zhihui Li

**Affiliations:** 1 Medical School of Chinese PLA, 28 Fuxing Road, Haidian District, Beijing, 100853 China; 2 Department of Ultrasound, The Third Medical Centre of PLA General Hospital, 69 Yongding Road, Haidian District, Beijing, 100853 China; 3 Department of Ultrasound, Hainan Hospital of PLA General Hospital, 80 Jianglin Road, Haitang District, Sanya, 572013 China; 4 Vanke School of Public Health, Tsinghua University, Beijing, 100084 China; 5 Institute for healthy China, Tsinghua University, Beijing, 100084 China

**Keywords:** Randomized control trial, Iliac artery hemostasis, Resuscitative endovascular balloon occlusion of the aorta, Portable ultrasound

## Abstract

**Purpose:**

This study aimed at to comparing the effectiveness of portable ultrasound guided REBOA vs. traditional manual extracorporeal compression in stopping iliac artery hemostasis.

**Methods:**

Twelve swine were included in this study (treatment group vs. control group, 6:6). A biopsy device was used to create an iliac artery rupture and hemorrhage in each swine. After 30 s of bleeding, the treatment group received REBOA under the guidance of ultrasound, whereas the control group received traditional manual extracorporeal compression. General physiological conditions were recorded at 0 s (baseline, T1), 30 s (initiation of therapies to stop bleeding, T2), 10 min (T3) and 30 min (T4) after bleeding. Intraperitoneal and retroperitoneal hemorrhage and specimens of iliac artery were collected after all swine were euthanized.

**Results:**

One swine was excluded because of accidental death not related to the experiment; thus, 11 swine were analyzed in this study. The general physiological characteristics of the two groups showed no difference at T1. Hemorrhagic shock occurred in both groups. After the hemostatic procedure was performed, systolic pressure, diastolic pressure and heart rate first increased significantly between T2 and T3, and then became stable between T3 and T4; these indicators in the control group deteriorated over time. The total blood loss in the treatment group (1245.23 ± 190.07 g) was much significantly less than that in the control group (2605.63 ± 291.67 g) with *p* < 0.001.

**Conclusions:**

Performing REBOA under the guidance of portable ultrasound is an effective way to stop bleeding. It suggests a potential alternative method for iliac artery hemostasis in the pre-hospital setting.

**Supplementary Information:**

The online version contains supplementary material available at 10.1007/s00068-022-01895-1.

## Background

Junctional hemorrhage, which refers to a hemorrhage that occurs at the junction of an extremity with the torso at an anatomic location, is caused by abdominopelvic, thoracic and lower cervical injuries [1]. It is a primary cause of death due to trauma because of the poor effectiveness in controlling bleeding with traditional methods, especially in the pre-hospital settings [2]. A study on U.S. combat fatalities showed that, between 2001 and 2011, 17.5% (171/976) of potentially preventable pre-hospital deaths were caused by junctional hemorrhage [3]. Another study showed a mortality rate of 45% among patients with junctional hemorrhage in the pre-hospital setting [4].

As a traditional hemostatic method, compression with standard gauze is widely used in many pre-hospital and transportation settings; however, a low survival rate of 12–55% has been shown in previous studies [5–9]. Recent studies have reported applying new hemostatic devices, such as AAJTTM, CRoCTM, JETTTM, SAM-JTTM to treat junctional hemorrhage in the pre-hospital and transportation settings [10–13]. However, a previous study that evaluated their effectiveness showed poor performance of these devices in terms of application time and pulse elimination[14]. Resuscitative endovascular balloon occlusion of the aorta (REBOA) was initial proposed by Hughes, and first used for injured soldiers in the Korean war [1]. As a minimally invasive procedure, progressively growing interest in REBOA has arisen, as it can be used for temporary hemostasis as an emerging strategy in many clinical settings [15, 16]. REBOA has been shown to provide promising support for life-threating hemorrhage patients with less mortality. A study showed superior survival outcomes with REBOA compared to resuscitative thoracotomy with aortic cross-clamping in patients with severe hemorrhagic shock (mortality rate, 62.5% vs. 16.7%, *p* < 0.001) [16]. A retrospective study on trauma management also showed that REBOA was significantly associated with lower mortality (adjusted odds ratio of survival, 7.4; 95% CI, 1.1–51.1) [17]. However, the effectiveness of REBOA in the pre-hospital setting is still controversial as it largely depends on the accuracy and punctuality of placing the balloon. A study showed that 6 of 19 REBOA attempts (32%) in trauma patients with exsanguinating pelvic hemorrhage failed in the pre-hospital setting, mainly due to inaccuracies in locating the bleeding site or placing the balloon [18]. Consequently, we assume that the aid of an imaging technique could be beneficial for the use of REBOA.

Using fluoroscopy or CT to guide the process of REBOA is popular among clinical patients [11, 12], yet these imaging instruments are not feasible in the pre-hospital setting due to their large size and inconvenient operation. However, the usage of portable ultrasound in the pre-hospital setting has gained increasing attention during the past decade [19]. Although many studies have shown the effectiveness of using ultrasound to evaluate bleeding after intraperitoneal trauma, to diagnose and grade abdominal organ trauma, and to guide the injection of hemostatic agents [14–16], no previous study has revealed the effects of using potable ultrasound to guide REBOA for junctional hemorrhage. In this study, we explored the effectiveness of applying portable ultrasound to guide REBOA for iliac artery hemostasis in the first aid pre-hospital setting.

## Materials and methods

### Ethics

All experiments were conducted following the Guide for the Care and Use of Laboratory Animals, approved by the Institutional Animal Care and Use Ethics Committee of China, and guidelines for the care of laboratory animals issued by the National Institutes of Health [license number, SYXK (Beijing) 2007-004]. All swine in this study were given a 72-h period to acclimate to the experimental facilities before the experiment.

### Main instruments

Ultrasound examination and guidance were performed by using a mobile CX50 system (Philips Medical Systems, Andover, MA). Both L9-3 and C5-1 transducer with 3–9 MHz and 1–5 MHz were used in this study. Blood pressure was monitored invasively using a TranStar^R^ Single Monitoring Kit (Smiths Medical ASD, Inc. Dublin, USA) and TranStar^R^ Pressure Monitoring System. MC1816 MaxCore disposable biopsy device with a diameter of 1.2 mm and a length of 16 cm was merchandised from Bard Company (NJ, USA to create iliac artery rupture (Appendix Fig. 1). The aortic balloon was placed using an Armada 35/Armada 35 LL PTA catheter (Abbott Molecular, De Plaines,IL,USA) (Appendix Fig. 2).

### Study design

This experiment thre following followed four steps of the procedure: (1) animal preparation, (2) randomization of the animals, (3) modeling iliac artery trauma, and (4) therapies to stop bleeding. A detailed description is provided below.

#### Animal preparation

**Fig. 1:**
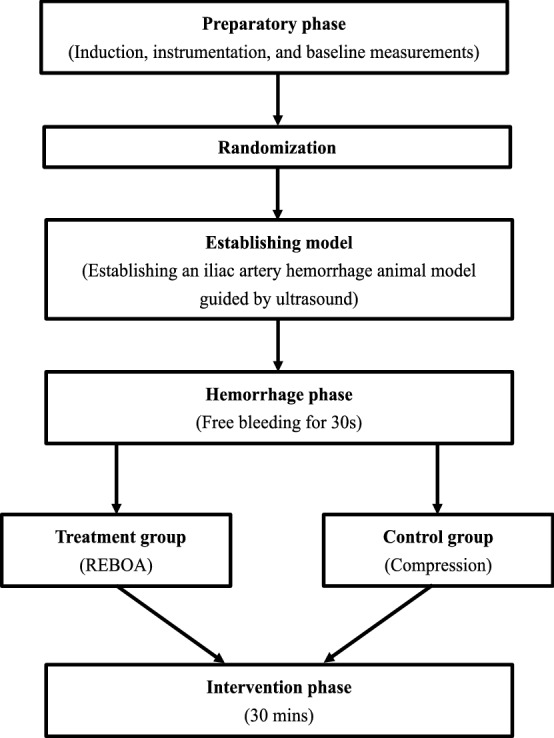
Study Schema

Twelve healthy Wuzhishan swine were included in this study. Basic anesthesia was induced by an intramuscular injection of 0.1 ml/kg with a mixture of the Zoletil^®^50 (Virbac, Carros, France) and Sumianxin II (Military Veterinary Institute, China) at a ratio of 1:2. Swine were placed in the supine position on the laboratory bench. The hair in the cervical, belly and groin regions was removed for ultrasound and cannulation. Under the guidance of portable ultrasound, we inserted a central venous catheter percutaneously into the right internal jugular vein to maintain anesthesia with 1/3 of the initial drug dose, depending on the animal’s conscious state. We cannulated the left common carotid artery under the guidance of ultrasound to monitor arterial pressure by the TranStar^R^ Single Monitoring Kit. Vital signs were monitored continuously through the TranStar^R^ Pressure Monitoring System. The study schema is shown in Fig. 1.

#### Randomization of the animals

Twelve numbered swine were randomly allocated to the control and treatment groups (6:6) according to a computer-generated random number table.

#### Modeling iliac artery trauma

The swine were deeply anesthetized, and the left iliac arteries was the injury target. The modeling procedure had three steps. First, the location of left iliac artery was confirmed by conventional ultrasound and was marked on the body surface. Second, the needle tip of the biopsy device was injected into the anterior lateral wall of the iliac artery at a 45–60° angle under the guidance of ultrasound. Third, the biopsy device was triggered and the iliac artery was punctured (See Additional file 1). A successful model had the following four characteristics: (1) the blood pressure decreased; (2) the heart rate first increased and then slowed down; (3) the iliac artery collapsed with a narrowed internal diameter; (4) the blood collections in the abdominal and retroperitoneal cavities increased. The procedure to model iliac artery trauma in all swine was performed by the same registered physician.

#### Therapies to stop bleeding

The treatment group received REBOA guided by portable ultrasound for iliac artery hemostasis, while the control group received traditional manual compression with dry gauze. Following previous practice, we started therapies for both groups after 30 s of free bleeding [20]. The procedures in both the treatment and control groups were done by one registered sonographer and one registered physician with 10 years of medical experience or more.

REBOA in the treatment group proceeded in five steps. First, the location of the right femoral artery (contralateral side) was confirmed by ultrasound and was marked on the body surface. Second, the right femoral artery was injected and catheterized with a sheath tube using the Seldinger technique under the guidance of potable ultrasound [21]. Third, the endovascular balloon was placed at the inferior segment of the abdominal aorta from the right femoral artery. Fourth, the endovascular balloon was inflated to occlude the aortic cavity once the injected liquid reached a certain quantity and pressure. Finally, two-dimensional ultrasound was used to visualize the proper place of the aorta balloon and Doppler ultrasound was used to verify the loss of aorta flow (Appendix Fig. 3).

For the control group, we first used ultrasound to evaluate the location of the left femoral artery (affected side), and then placed dry gauze on the body surface of the affected iliac artery. Manual extracorporeal compression was performed and ultrasound was used to visualize the ipsilateral femoral artery flow.

### Main outcome measures

Our study included five outcome measures, including systolic pressure (SP), diastolic pressure (DP), heart rate (HR), the maximum depth of the ascites and total blood loss.

Systolic pressure (SP), diastolic pressure (DP) and heart rate (HR) were recorded in real-time using a TranStar^R^ Pressure Monitoring System at baseline (0 s, T1), 30 s (initiation of therapies to stop bleeding, T2), 10 min (T3) and 30 min (T4) after bleeding started. The maximum depth of the intraperitoneal and retroperitoneal blood collections was measured by ultrasound at the same time.

Once all animals were killed at the end of T4, the bleeding clots from the intraperitoneal and retroperitoneal cavity were measured by the weight of the blood-soaked gauze. Gross specimens of the abdominal aorta and both the iliac arteries were collected.

### Statistical analysis

We performed a description of data using mean values and standard deviations (SD). We checked the homogeneity of all swine at baseline and the amount total blood loss between the two groups using *t *tests. To determine the statistical significance of differences in the outcome indicators (SP, DP, HR, and maximum depth of anechoic zone) between the treatment and the control groups, we adopted a repeated-measures ANOVA (RMANOVA) for analysis, with time as the within-group trend and treatment as the cross-group factor. To protect against Type I errors of RMANOVA, we collected the degrees of freedom using the Greenhouse-Geisser method, in which sphericity assumptions were not upheld. A significant group*time interaction term suggested that the treatment group and the control group were different in terms of changing trends; post-hoc analysis was performed using one-way ANOVA at each time point in the within-group comparison. All analyses were carried out using SPSS software (version 19.0 for windows; IBM co., NY, USA; serial #: 5,087,722). A *p* value < 0.05 was considered statistically significant.

## Results

### Baseline characteristics

We recruited 12 swine for this study; however, one swine in the control group was excluded due to accidental death unrelated to the experiment. Thus, 11 animals were enrolled in the study. At baseline, there was no significant difference in any of the general physiological conditions between the two groups, including weight (14.08 ± 1.28 kg vs.14.10 ± 0.10 kg, *p* = 0.541), abdominal aortic diameter (0.69 ± 0.05 cm vs. 0.65 ± 0.06 cm, *p* = 0.475), SP (121.17 ± 11.51 mmHg vs. 118.40 ± 12.34 mmHg, *p* = 0.710), DP (91.50 ± 12.05 mmHg vs. 87.80 ± 15.55 mmHg, *p* = 0.712), HR (80.83 ± 6.11 bpm vs. 82.40 ± 4.28 bpm, *p* = 0.642), and the maximum depth of the ascites (0.07 ± 0.08 cm vs. 0.06 ± 0.09 cm, *p* = 0.820). These results are shown in Table 1.

**Table 1:** Baseline characteristics in both groups (Mean ± SD)

Group	Number	Weight (kg)	Abdominal aortic diameter (cm)	Systolic pressure (mmHg)	Diastolic pressure (mmHg)	Heart rate (bpm)	Maximum depth of the ascites (cm)
Treatment group	6	14.08 ± 1.28	0.69 ± 0.05	121.17 ± 11.51	91.50 ± 12.05	80.83 ± 6.11	0.07 ± 0.08
Control group	5	14.10 ± 0.10	0.65 ± 0.06	118.40 ± 12.34	87.80 ± 15.55	82.40 ± 4.28	0.06 ± 0.09
*P* values		0.541	0.475	0.710	0.712	0.642	0.820

### The effects of modeling iliac artery trauma

We show the measurements of SP, DP, HR, and maximum depth of the ascites in Table 2. Hemorrhagic shock occurred in both treatment and control groups after the left iliac artery was wounded. Between T1 and T2, SP and DP reduced significantly in both the treatment and the control groups, while HR and maximum depth of the ascites significantly increased. The changes in SP, DP, HR, and maximum depth of the ascites during T1 and T2 showed no significant differences between the two groups. SP in the treatment group significantly fell from 121.17 ± 11.51 mmHg at T1 to 83.00 ± 16.64 mmHg at T2 (*p* < 0.001). As for the control group, SP dropped from 118.40 ± 12.34 mmHg at T1 to 85.40 ± 13.65 mmHg at T2 (*p* < 0.001). There was no obvious difference in the changing trend from T1 to T2 between the two groups (Table 2 and Appendix Table 1).

**Table 2:** The changes in hemodynamic and physiological parameters in G1 and G2 at different times

Group/time	Systolic pressure (mmHg)	Diastolic pressure (mmHg)	Heart rate (bpm)	Maximum depth of the ascites (cm)
Treatment group				
T1	121.17 ± 11.51	91.50 ± 12.05	80.83 ± 6.11	0.07 ± 0.08
T2	83.00 ± 16.64	60.17 ± 16.76	101.50 ± 5.39	2.32 ± 0.25
T3	97.17 ± 11.92	66.67 ± 12.34	111.83 ± 7.39	3.10 ± 0.41
T4	97.50 ± 9.29	67.67 ± 10.78	113.83 ± 5.49	3.50 ± 0.36
Control group				
T1	118.40 ± 12.34	87.80 ± 15.55	82.40 ± 4.28	0.06 ± 0.09
T2	85.40 ± 13.65	56.20 ± 7.40	103.20 ± 3.70	2.46 ± 0.25
T3	71.20 ± 6.18	43.80 ± 5.63	122.00 ± 4.53	4.12 ± 0.24
T4	62.40 ± 3.44	35.00 ± 3.39	132.40 ± 3.98	5.14 ± 0.35
*P* time (*F* time)	< 0.001 (145.103)	< 0.001 (89.259)	< 0.001 (549.658)	< 0.001 (990.583)
*P* group (*F* group)	0.040 (5.732)	0.031 (6.547)	0.023 (7.419)	0.001 (27.130)
*P* time * group (*F* time * group)	< 0.001 (34.081)	< 0.001 (16.121)	< 0.001 (26.597)	< 0.001 (42.510)

*T1* baseline, *T2* free bleeding for the 30 s (start performing therapies to stop bleeding), *T3* 10 min after bleeding, *T4* 30 min after bleeding, time * group the differences on hemodynamic and physiological parameters between 2 groups over time

### Comparing within-group trends and cross-group differences between the treatment and control groups

After 30 s of free bleeding, the treatment group received REBOA guided by portable ultrasound, while the control group received traditional manual compression with dry gauze.

In the treatment group, SP and DP increased to 97.17 ± 11.92 mmHg (T2 vs. T3, *p* = 0.040) and 66.67 ± 12.34 mmHg (T2 vs. T3, *p* = 0.214) at T3, respectively. HR and the maximum depth of the ascites also increased to 111.83 ± 7.39 bpm (T2 vs. T3, *p* = 0.001) and 3.10 ± 0.41 cm (T2 vs. T3, *p* = 0.001) at T3. Between T3 and T4, the measurements of SP, DP, and HR remained stable, which were 97.50 ± 9.29 mmHg (T3 vs. T4, *p* = 0.846), 67.67 ± 10.78 mmHg (T3 vs. T4, *p* = 0.518), and 113.83 ± 5.49 bpm (T3 vs. T4, *p* = 0.352) at T4, respectively. The maximum depth of the ascites slightly increased to 3.50 ± 0.36 cm at T4 (T3 vs. T4, *p* < 0.001) (Table 2 and Appendix Table 1).

In the control group, SP fell from 85.40 ± 13.65 mmHg at T2 to 71.20 ± 6.18 mmHg at T3 (T2 vs. T3, *p* = 0.016), and continued drop to 62.40 ± 3.44 mmHg at T4 (T3 vs. T4, *p* = 0.003). DP showed similar trend as SP. HR increased significantly from 103.20 ± 3.70 bpm at T2 to 122.00 ± 4.53 bpm at T3 (T2 vs. T3, *p* < 0.001), and arose to 132.40 ± 3.98 bpm at T4 (T3 vs. T4, *p* < 0.001). The maximum depth of the ascites kept increasing from 2.46 ± 0.25 cm at T2 to 4.12 ± 0.24 cm at T3 (T2 vs. T3, *p* < 0.001), and further to 5.14 ± 0.35 cm at T4 (T3 vs. T4, *p* < 0.001; Table 2 and Appendix Table 1).

**Fig. 2:**
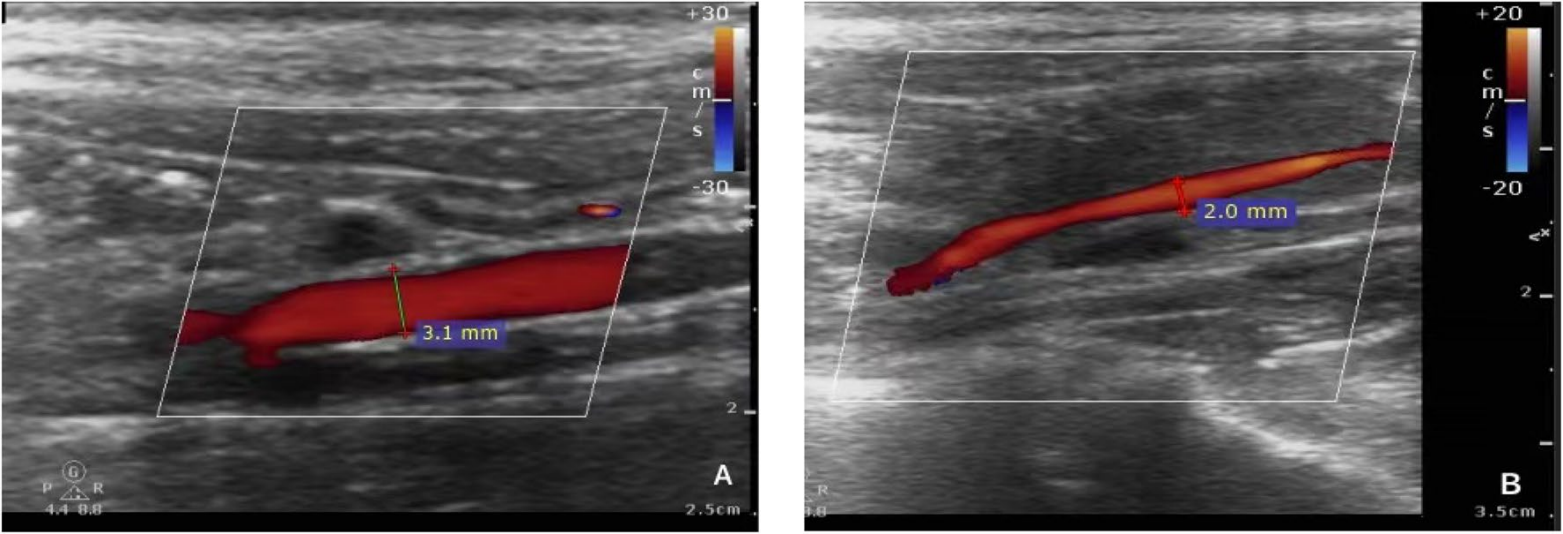
Changes in iliac artery blood flow before and after modeling. **A**: Before modeling, the internal diameter of the iliac artery was 0.31 cm. Color Doppler ultrasound showed the blood flow of the iliac artery. **B**: After free bleeding for the 30 s, color Doppler ultrasound showed that the artery rapidly collapsed with less blood flow and the inner diameter of the artery narrowed. The internal diameter of the iliac artery was 0.20 cm. And color Doppler ultrasound showed that the iliac vein was compressed and there was no blood flow signal

The two groups appeared to be significantly different at T4 for all outcome, as suggested by the main effect of group in Table 2. The RMANOVA of DP revealed a main effect of group with F = 6.547 (*p* = 0.031). The interaction term between time and group also appeared to be significant for all outcomes, suggesting that the changing trends of the indicators over time were different across the two groups (Fig. 2).

### Ultrasound imaging

**Fig. 3:**
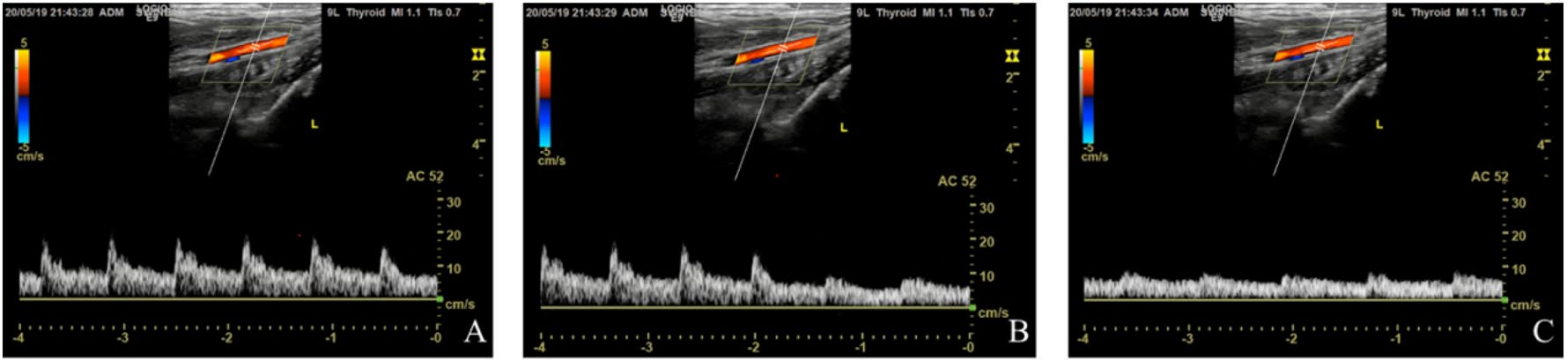
After the balloon inflated at the abdominal aorta, the right iliac artery was disappeared. **A**: It was the normal Doppler spectrum of the right iliac artery. **B**: With the balloon was inflating, the Doppler spectrum of the right iliac artery was changed. **C**: After the balloon inflated, the Doppler spectrum of the right iliac artery was disappeared

Two-dimensional ultrasound showed iliac artery collapse in all swine and a rapidly decreased inner diameter. Color Doppler showed decreased blood flow in the iliac artery, and the blood velocity also decreased, as shown by spectrum Doppler. We also found that the iliac vein was compressed and there was no blood flow signal in it (Fig. 3).

When the balloon catheter was moved from the contralateral side (right femoral artery) to the lower abdominal aorta under ultrasound guidance in the treatment group, the balloon was inflated (See Additional file 2). The blood flow of the distal abdominal aorta and iliac artery reduced or disappeared immediately, as shown by color Doppler (Appendix Fig. 4 and See Additional file 3). Spectrum Doppler showed the aorta had been occluded completely with the balloon. When the balloon was collapsed, the blood flow of the distal abdominal aorta and iliac artery occurred rapidly to avoid ischemia in the distal extremities. The blood collections in the abdominal and retroperitoneal cavities was imaged as a large number of anechoic regions at T2, which increased slowly in the treatment group but increased rapidly in the control group at T3 and T4, shown by real-time ultrasound (Appendix Fig. 5).

### Pathological findings

All swine were dissected after being culled, and numbers of blood clots in the abdominal and retroperitoneum were determined. Gross pathology confirmed the rupture of the left iliac artery (Appendix Fig. 6). Total blood loss was measured by quantifying blood clots and blood-soaked gauze; a statistically significant difference was found in the total blood loss at T1 (1245.23 ± 190.07 g) and T2 (2605.63 ± 291.67 g), with *p* = 0.002.

## Discussion

In this randomized control animal trial, we obtained two salient findings. First, it is feasible and effective to use portable ultrasound to guide REBOA in the first aid pre-hospital setting. REBOA under the guidance of potable ultrasound showed a success rate of 100% (6/6) in improving health outcome measurements (i.e., SP, DP, HR, maximum depth of ascites, and total blood loss). Second, we found that the treatment group receiving REBOA under the guidance of ultrasound had significantly better health outcomes than the control group that received traditional manual extracorporeal compression. For example, the measured SP in the treatment group was higher than in the control group with a large difference of 35 mmHg at 30 min after bleeding started; the total blood loss was 1.2 kg in the treatment group, compared to 2.6 kg in the control group.

Our result on the feasibility and effectiveness of using imaging technology to guide intra-arterial placement of REBOA was consistent with the previous studies [22, 23]. Viktor et al. [24] compared the effectiveness of ultrasound-guided puncture of the femoral artery during REBOA with blind puncture in the pre-hospital setting. The results showed that only 1 out of 6 blind punctures was successful, whereas 8 of 9 ultrasound-guided punctures was successful and ultrasound-guided puncture of the femoral artery took only 65 s on average (5–260 s). Brede et al. [25] performed ultrasound-guided REBOA in 10 patients with non-traumatic cardiac arrest on a helicopter. The success rate of the first insertion of REBOA was as high as 80%.

Portable ultrasound, including hand-held ultrasound and palmar ultrasound, is a critical instrument to the success of REBOA in the pre-hospital setting. Besides being easy to carry and non-invasive, portable ultrasound plays a key role in providing timely support to the procedure. It is worth noting that REBOA has a strongly time-sensitive nature [26]. When REBOA blocks the junction of the iliac artery for more than 80 min, it can cause severe vascular paralysis, systemic inflammation and lactic acidosis, leading to potentially fatal ischemia of the trunk, viscera and spinal cord [14, 27, 28]. Markov et al. [24] observed that blood lactate increased after REBOA in the porcine trauma model at different periods, and the level of increase was proportional to the time of blockage. Moreover, aortic occlusion for more than 90 min could lead to organ dysfunction, including renal insufficiency and hepatic lobular necrosis. Given these characteristics, minimizing delays is the most important factor related to the success of REBOA. Portable ultrasound has shown great potential in this procedure [29].

There are a few limitations to this study. Firstly, the iliac artery hemorrhage was performed using a biopsy device, which might be a little different from the closed trauma that occurs in patients. Secondly, although portable ultrasound guidance allowed for real-time visualization of balloon [24] implantation and improved accuracy and safety compared with the traditional method of gauze compression, but there was a greater dependence on technology in the treatment group than in the control group [24]. It is necessary that the operator is trained in ultrasound diagnosis and interventional puncture. Third, whether the results generated from swine could be applied to other animals or human beings need further investigation. Therefore, further studies are needed regarding the extent of ischemia-reperfusion injury to the limbs and the severity of interruption to distal abdominal organs at different time points after REBOA.

## Conclusions

Despite some limitations, our study demonstrates that using portable ultrasound-guided REBOA to treat iliac artery hemostasis in severe trauma generates superior results than the traditional method in the pre-hospital setting. Further studies should be done to investigate its effect on other animals and humans.

## Supplementary Information

Below is the link to the electronic supplementary material.

Supplementary file1 (DOCX 3710 KB)

## Data Availability

The datasets used and/or analyzed during the current study are available from the corresponding author on reasonable request.
