# Supplementary material for: The effectiveness of portable ultrasound-guided resuscitative endovascular balloon occlusion of the aorta for stopping iliac artery hemorrhage during first aid pre-hospital: a randomized control animal trial
Source: Eur J Trauma Emerg Surg. 2022 Apr 12;48(4):2841–8. doi: 10.1007/s00068-022-01895-1 (PMC9360081; doi:10.1007/s00068-022-01895-1)
Supplement: Supplementary file 1 — Supplementary file1 (DOCX 3710 KB) [file 68_2022_1895_MOESM1_ESM.docx]

**Additional file**

**Table 1.** Compared with the previous time point in the within-group by using one-way ANOVAs.

**Fig. 1.** MaxCore disposable puncture biopsy device used in modeling.

**Fig. 2.** The balloon used for aorta occlusion.

**Fig. 3.** The iliac artery was punched and the balloon was placed under ultrasound-guided.

**Fig. 4.** Comparison of the maximum depth of the anechoic zone in the abdominal cavity between the two groups at 30min.

**Fig. 5.** Pathological images of right lower extremity muscle tissue before experiment and bleeding for 30min.

**Fig. 6.** The gross specimen of the common iliac artery and huge blood clots.

**Table 1. Compared with the previous time point in the within-group by using one-way ANOVAs**

| Group/*P* values | Systolic Pressure (mmHg) | Diastolic Pressure(mmHg) | Heart Rate (bpm) | Maximum Depth of Ascites  (cm) |
| --- | --- | --- | --- | --- |
| **Treatment group** |  |  |  |  |
| *P* (T1 vs T2) | <0.001 | <0.001 | <0.001 | <0.001 |
| *P* (T2 vs T3) | 0.040 | 0.214 | 0.001 | 0.001 |
| *P* (T3 vs T4) | 0.846 | 0.518 | 0.352 | <0.001 |
| **Control group** |  |  |  |  |
| *P* (T1 vs T2) | <0.001 | 0.004 | <0.001 | <0.001 |
| *P* (T2 vs T3) | 0.016 | <0.001 | <0.001 | <0.001 |
| *P* (T3 vs T4) | 0.003 | 0.001 | <0.001 | 0.001 |

**Fig. 1.** MaxCore disposable puncture biopsy device used in modeling.


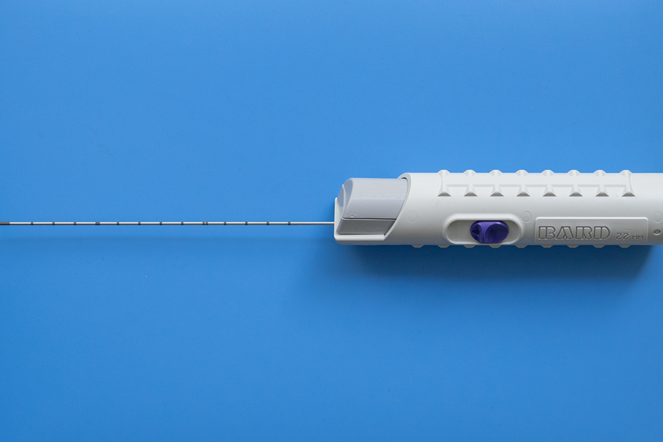


**Fig. 2.** The balloon used for aorta occlusion.


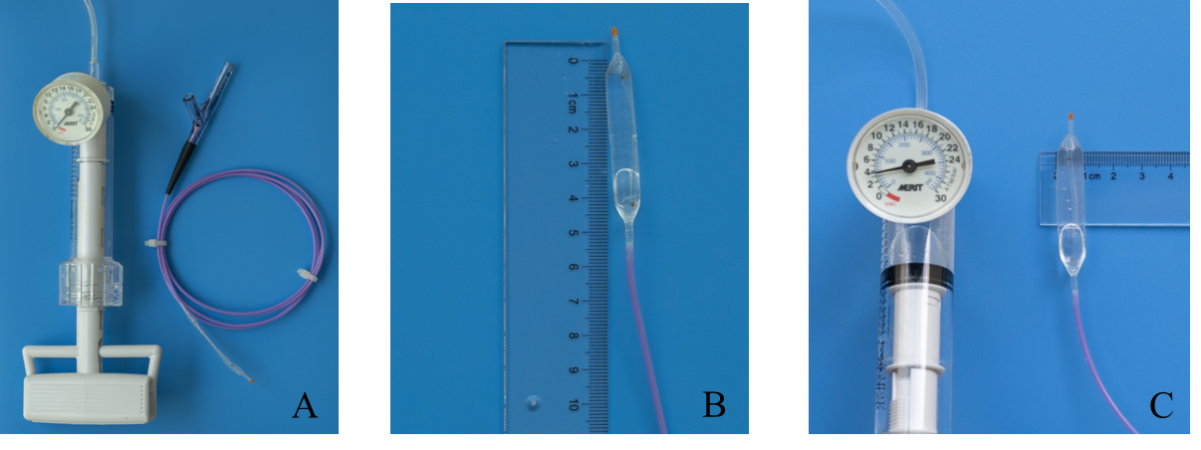


A: The endovascular balloon of aorta occlusion and pressure pump.

B: The length of the balloon is about 60 mm.

C: When the pressure inside the balloon is 4 ATM (standard atmospheric pressure), the diameter of the balloon is 10 mm.

**Fig. 3.** The iliac artery was punched and the balloon was placed under ultrasound-guided.


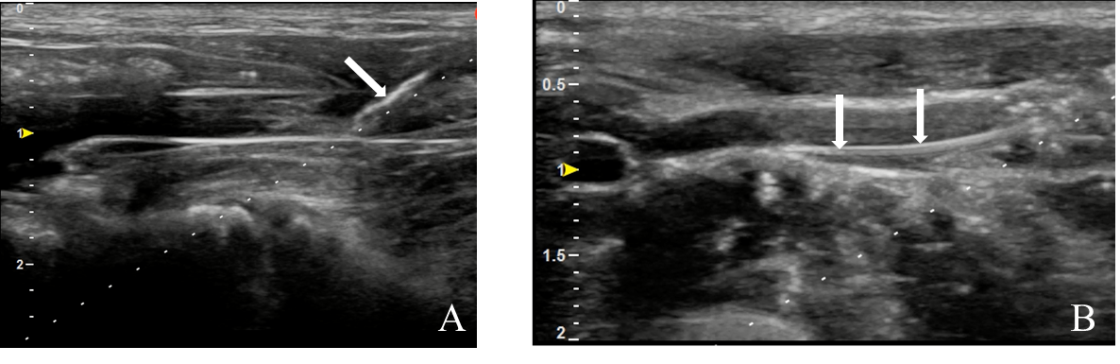


A: Under ultrasound guidance, an endovascular balloon was placed at the lower abdominal aorta via the Seldinger technique. The arrow points to the needle tip.

B: The guidewire (dash line) is carried along the iliac artery. The arrow points to the balloon pipe.

**Fig. 4.** Comparison of the maximum depth of the anechoic zone in the abdominal cavity between the two groups at 30min.


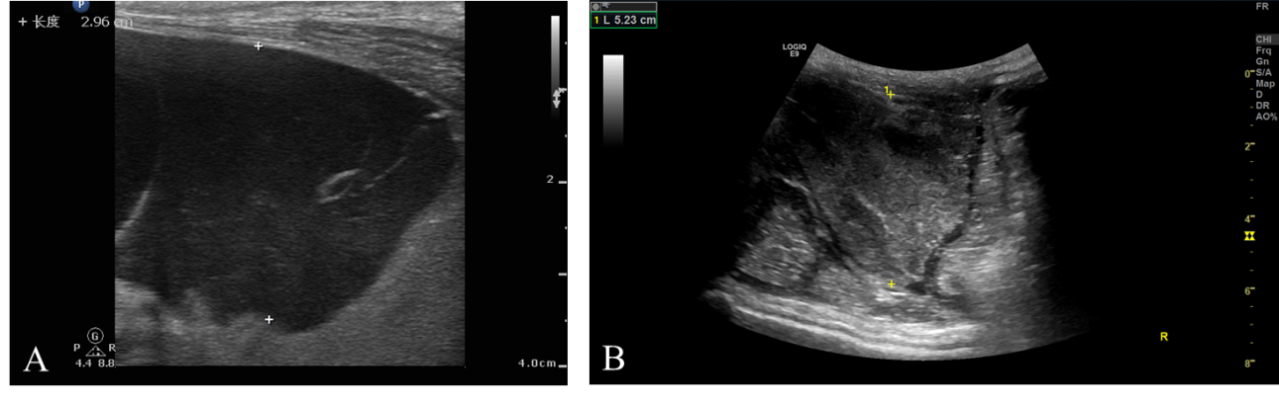


A: With the time of balloon inflation, the maximum depth of the anechoic zone in the abdominal cavity was increased slowly (2.96cm).

B: In the control group, the maximum depth of the anechoic zone in the abdominal cavity was still increasing under continuous pressure of gauze (5.23cm).

**Fig. 5.** Pathological images of right lower extremity muscle tissue before experiment and bleeding for 30min.


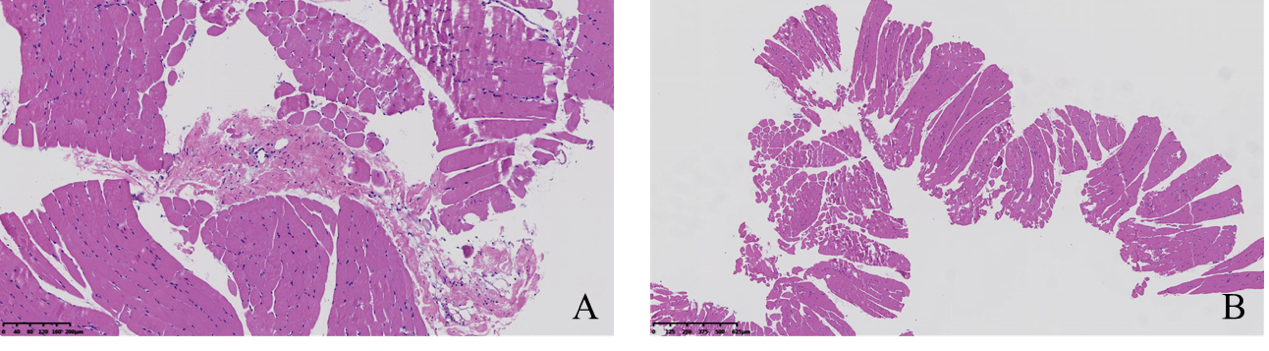


A: The histopathological image of the right lower limb muscle before the experiment (10x).

B: In the intervention group, no significant changes were observed in the same part of the right lower limb when bleeding for 30min (4x).

**Fig. 6.** The gross specimen of the common iliac artery and huge blood clots.


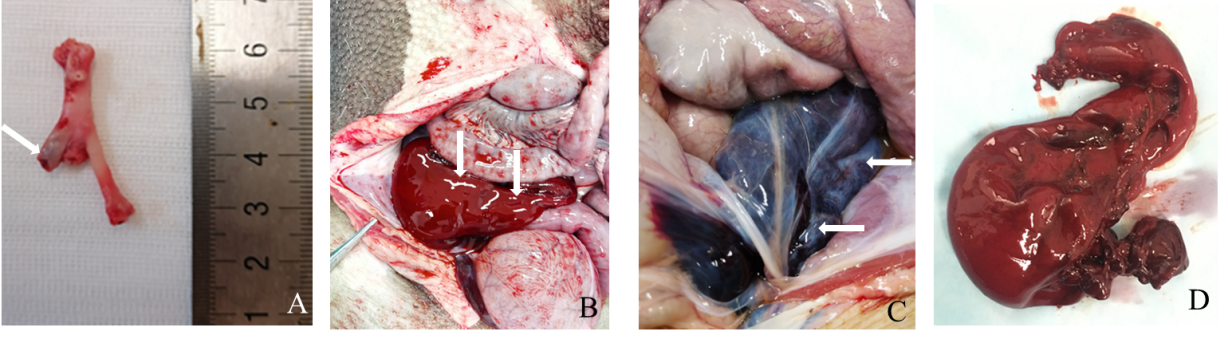


A: The gross specimen of the abdominal aorta and bilateral iliac artery. The arrow points to the rupture of the common iliac artery.

B: After the animals were sacrificed and the abdominal was opened, huge blood clots in the abdominal cavity were visible (arrows).

C: Huge blood clots in the retroperitoneum were visible (arrows).

D: One of the abdominal cavity blood clots.
